# Cardiac pacing and lead devices management: 25 years of research at EP Europace journal

**DOI:** 10.1093/europace/euad202

**Published:** 2023-07-08

**Authors:** Pascal Defaye, Mauro Biffi, Mikhael El-Chami, Serge Boveda, Michael Glikson, Jonathan Piccini, Marco Vitolo

**Affiliations:** Cardiology Department, University Hospital and Grenoble Alpes University, CS 10217, Grenoble Cedex 9, Grenoble 38043, France; Cardiology Unit, Cardiac Thoracic and Vascular Department, IRCCS Azienda Ospedaliero-Universitaria di Bologna, Bologna, Italy; Department of Medicine, Division of Cardiology, Emory University School of Medicine, Atlanta, Georgia, USA; Clinique Pasteur, Heart Rhythm Department, Toulouse, France; Cardiology Department, Jesselson Integrated Heart Center Shaare Zedek Medical Center and Hebrew University Faculty of Medicine, Jerusalem, Israel; Duke University, Duke Clinical Research Institute, Durham, NC, USA; Cardiology Division, Department of Biomedical, Metabolic and Neural Sciences, University of Modena and Reggio Emilia, Policlinico Di Modena, Modena, Italy; Clinical and Experimental Medicine PhD Program, University of Modena and Reggio Emilia, Modena, Italy

**Keywords:** Pacemaker, Cardiac pacing, CIED, Leadless pacing, State of the art

## Abstract

**Aims:**

Cardiac pacing represents a key element in the field of electrophysiology and the treatment of conduction diseases. Since the first issue published in 1999, EP Europace has significantly contributed to the development and dissemination of the research in this area.

**Methods:**

In the last 25 years, there has been a continuous improvement of technologies and a great expansion of clinical indications making the field of cardiac pacing a fertile ground for research still today. Pacemaker technology has rapidly evolved, from the first external devices with limited longevity, passing through conventional transvenous pacemakers to leadless devices. Constant innovations in pacemaker size, longevity, pacing mode, algorithms, and remote monitoring highlight that the fascinating and exciting journey of cardiac pacing is not over yet.

**Conclusion:**

The aim of the present review is to provide the current ‘*state of the art*’ on cardiac pacing highlighting the most important contributions from the Journal in the field.

## Introduction

Cardiac pacing represents a key element in the field of electrophysiology and the treatment of conduction diseases. Since the first issue published in 1999, EP Europace has significantly contributed to the development and dissemination of the research in this area.^1^ In the last 25 years, there has been a continuous improvement of technologies and a great expansion of clinical indications making the field of cardiac pacing a fertile ground for research still today. Several issues have been an object of debate and subject of extensive research. Starting from 1999, more than 1 300 papers focused on several different aspects of cardiac pacing have been published in the Journal. The aim of the present review is to provide the current ‘*state of the art*’ on cardiac pacing highlighting the most important contributions from the Journal in the field.

### History of cardiac pacing: a long and fascinating journey

The history of cardiac pacing is a long and fascinating journey with distant origins.^2,3^ In the late 1700, the Italian physician Luigi Galvani published his first experimental findings describing the effect of an electric current on the muscles of dead frogs’ legs and heart laying the ground for modern cardiac electrophysiology.^2^ Early attempts to artificially pace the human heart began in the 1930s with the pioneering experiences of the Australian anaesthetist Mark Lidweel and the American physiologist Albert S. Hyman in the setting of cardiac resuscitation.^4^ Albert S. Hyman first reported a “*resuscitation of the stopped heart by intracardial therapy*” by the “*experimental use of an artificial pacemaker*” coining the term we still used today.^2,5^ In the 1950s and the early 1960s, the historical experiences of Wilfred Bigelow, John Callaghan, Jack Hopps, and Paul M. Zoll later paved the way for the development of the pacing technology and the clinical application of pacemakers to treat cardiac arrhythmias.^2^

The first fully implantable pacemaker was performed in Stockholm in 1958 by the cardiac surgeon Åke Senning, using a device built by the medical engineer Rune Elmqvist. The device was successfully implanted in Arne Larsonn, a 43-year-old patient who suffered from Stokes–Admans attacks secondary a myocarditis. The first device weighed 180 g (compared to 20–50 g of modern pacemakers), and the pulse generator failed within a few hours from the implant being replaced the same day.^2,3^ Arne Larsonn underwent 26 pacemaker replacements and died at age 86 from malignant skin cancer, a sign of the success of this technology.^2^ The implementation of permanent cardiac pacing in clinical practice was firstly aimed at treating Morgagni–Adams–Stokes syndrome or bradyarrhythmic cardiac arrest, and only later was cardiac pacing specifically designed to treat different forms of bradyarrhythmia. After the initial breakthrough experience, the advances in pacemaker technology with the parallel increase of the clinical indications for pacing led to continuous improvements in the field.^6^ The technology has rapidly evolved, from the first external devices with limited longevity, passing through conventional transvenous pacemakers (TV-PPM) to leadless devices.^7^ Constant innovations in pacemaker size, longevity, pacing mode, algorithms, and remote monitoring highlight that this fascinating and exciting journey is not over yet.^6,8–11^

### Epidemiology of pacemaker implantations

In the last decades, the use of pacemakers has dramatically increased.^6,12–15^ From an epidemiological perspective, the ageing population and the improving survival among patients with heart diseases who potentially need a pacemaker led to a significant increase in implantation rates.^16^ Recent estimates reported that the number of patients undergoing pacemaker implantation has steadily increased up to an annual implant rate of 1 million devices.^16^

Patients aged 65 and over are rapidly growing, counting today more than 8% of people worldwide with future predictions estimating an even higher percentage in 2050.^17^ Cardiac rhythm disturbances and the degeneration of the cardiac conduction system are significantly more prevalent in elderly patients with approximately 80% of pacemaker implants occurring in patients older than 65 years old.^6,13,18,19^ In parallel, the most recent European Society of Cardiology (ESC) guidelines have expanded the indications for pacemaker implantation leading to a substantial increase in pacemaker use in different clinical settings.^6^

Nevertheless, a precise estimate of pacemaker implants is of difficult analysis since most of the data available derive from retrospective studies or real-world registries with their intrinsic typical limitations.^12^ A previous analysis of claims files from the Health Care Finance Administration for Medicare beneficiaries from 1990 to 1999 reported that rates of implantation of cardiac devices increased from 3.26 implantations per 1 000 beneficiaries in 1990 to 4.64 implantations per 1 000 beneficiaries in 1999, representing an increase of 42% in 10 years.^20^

**Figure 1:**
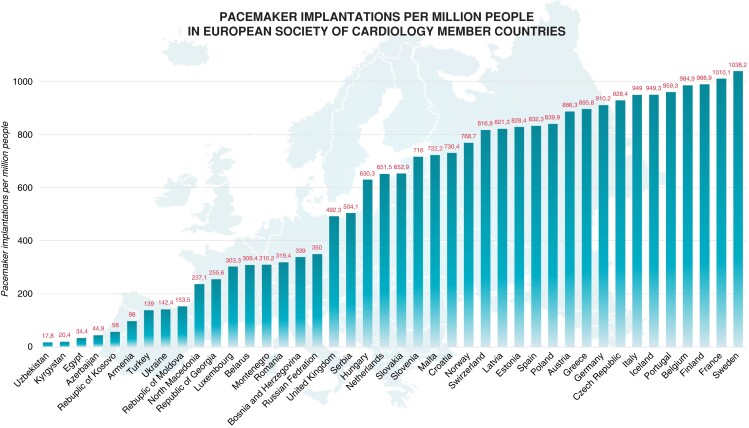
Pacemaker implantations per million people in European Society Cardiology member countries. Adapted from Timmis A. *et al*. European Society of Cardiology: cardiovascular disease statistics 2021. Eur Heart J. 2022.

According to the latest ESC cardiovascular disease statistics,^21^ there was a median of 652.2 (IQR 267.5–874.7) pacemaker implants per million inhabitants of ESC member countries (*Figure 1*). A significant variability of implant rates has been reported among countries, ranging from <50 pacemaker implantations per million people in Azerbaijan, Egypt, Kyrgyzstan, and Uzbekistan to >1 000 implantations per million people in France and Sweden.^21^ The 2020 survey of the ESC member countries reported that the median of hospitals implanting pacemakers per million people was 2.8 (IQR 1.7–4.4) with low performance in middle-income compared with high-income countries (<1 hospital per million people in Egypt, Kyrgyzstan, and Uzbekistan compared with >7 hospitals per million people in Belgium, Cyprus, Germany, and Switzerland) highlighting important geographical differences.^21^

### Modes and indications of cardiac pacing

**Figure 2:**
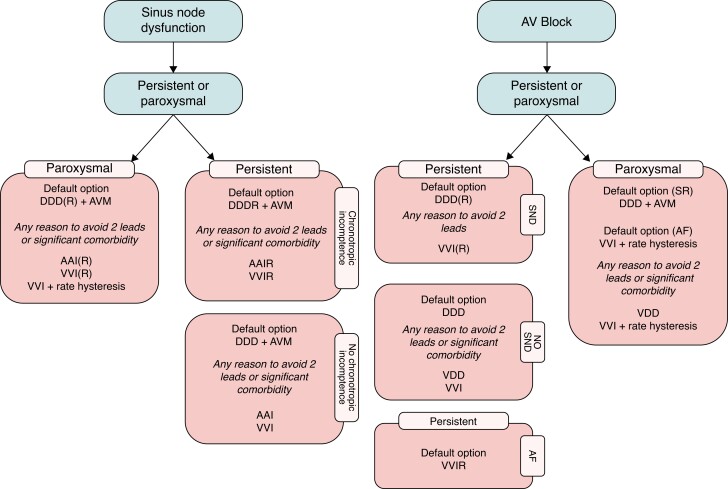
Optimal pacing mode in sinus node dysfunction and atrio-ventricular block. Adapted from ref.^6^ AF, atrial fibrillation; AV, atrioventricular; AVM, atrioventricular management [i.e. AV delay programming (avoiding values > 230 ms) or specific algorithms to avoid/reduce unnecessary ventricular pacing]; CRT, cardiac resynchronization therapy; SND, sinus node dysfunction; SR, sinus rhythm. (R) indicates that the programming of such a pacing mode is preferred only in the case of chronotropic incompetence. Reasons to avoid two leads include young age and limited venous access.

In general, the type and mode of cardiac pacing are determined by the specific nature of the conduction system disease [sinus node dysfunction (SND), atrioventricular (AV) block, bundle branch block, etc.].^6^ Usually, cardiac pacing is indicated in case of high-degree AV block or when the bradyarrhythmias are symptomatic.^6^*Figure 2* summarizes the optimal pacing mode in SND and AV block. A complete and detailed overview of types, modes, and indications for cardiac pacing has been recently reported in the latest 2021 ESC guidelines.^6^

### Optimal pacing mode and algorithm selection to avoid right ventricular pacing: current evidence and future directions

**Figure 3:**
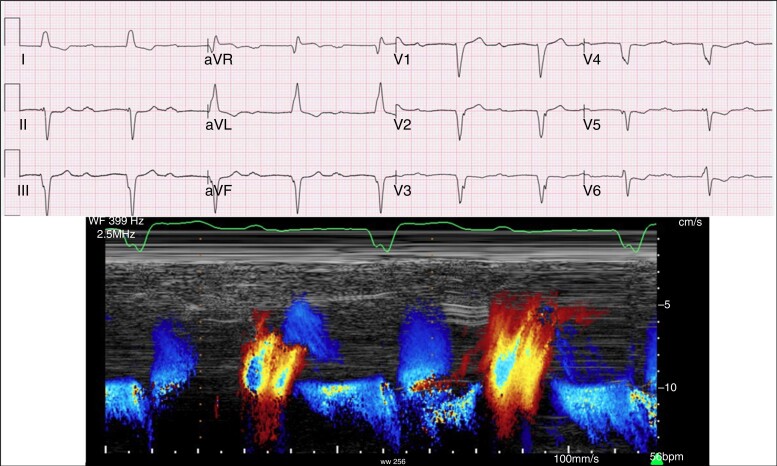
Example of a too-long PR interval enabled by the RVpm strategy, with severe mitral regurgitation at a PR interval = 560 ms.

Preservation of the physiologic cardiac activation from the atria to the ventricles is key to mimic the natural electromechanical coupling of the heart.^22,23^ Though enabling AV synchrony, DDD/R mode is burdened by about 24% incidence of persistent atrial fibrillation (AF) at 2 years in DDDR pacemaker recipients,^24^ and by a 12% prevalence of heart failure (HF).^25^ The cause of AF, left ventricular dysfunction, and HF is probably multifactorial and is until now incompletely understood,^22,24–26^ but to a certain extent, it is related to suboptimal AV coupling and the amount of right ventricular stimulation (RVp). The association of RVp > 30% with HF and AF development, hospitalizations, and death across multiple trials and clinical settings set the premises for the RVP minimization (RVpm) strategy, which prevents the unfavourable drawbacks of electro-mechanical dyssynchrony induced by RVp.^22^ To preserve the physiological ventricular activation, algorithms to minimize RVp were developed,^27^ whose functioning ranges from AV delay hysteresis with automatic search of intrinsic conduction (thereby determining 1:1 AV conduction) to automatic mode switching from DDD to AAI or ADI (which implies tolerance on non-conducted *P* waves). *Table 1* shows the key aspects of RVpm vs. maintenance of AV sequential stimulation in major clinical studies.^24,28–34^ An excellent review on the functioning of RVpm algorithms across manufacturers by Jankelson *et al.*^27^ highlights that AV delay search up to 450 ms provides the same extent of RVp reduction as ADI(AAI)/DDD switching algorithms in SND patients with AV block 1st, endpoints as AF burden, atrial volume, and LV function being similar in a randomized comparison,^35^ while the latter may be more effective in patients with intermittent AV block 2nd–3rd.^32^ Unwanted side effects of RVpm algorithms rarely consist of ventricular tachyarrhythmias determined by long pauses, whereas they most commonly are related to the occurrence of very long PR intervals, which may cause pacemaker-mediated tachycardia on one side or, in the worst of cases, AV uncoupling by an inefficient preload coupled to increased atrial pressure/stretching and sometimes functional mitral regurgitation (*Figure 3*). In fact, though earliest studies in SND patients with normal AV conduction proved that RVpm decreases persistent AF compared to customary DDD pacing, no survival benefit occurred.^22^ The broad population of pacemaker recipients is instead likely to have AV conduction shifting from normal to markedly prolonged (>300 ms) up to transient/permanent AV block owing to advanced age and changing medical conditions; thus, a trade-off between preserving the intrinsic cardiac activation and ensuring the optimal AV coupling becomes necessary.^22,27,36^ The ANSWER study^32^ used the RVpm strategy with a feature to pace also in the event of persistently long PR intervals in a mixed population (42% of intermittent AV block patients): a significant reduction of secondary endpoints (cardiac death/HF hospitalization and cardiovascular hospitalizations) occurred in the RVpm arm, hinting that RVpm is worthwhile but should allow physiological (<300 ms) AV intervals.^32^ The delicate balance of targeting these two endpoints came evident in several trials, which pinpointed a long PR interval as a risk marker for AF and HF in pacemaker recipients and implantable cardioverter–defibrillator (ICD) candidates.^22, 25,37,38^ Indeed, an increased incidence of AF at long term by the RVpm strategy occurred in SND patients with a baseline PR > 180 ms compared to maintenance of AV coupling by DDDR pacing in the DANPACE trial, which also found no difference in AF occurrence and burden based on the amount of RVp, while no difference was observed in terms of mortality, HF, AF, and stroke in the long-term between AAIR and DDDR pacing.^22,37,38^

**Table 1:** Key aspects of RVpm vs. maintenance of AV sequential stimulation in major clinical studies. Adapted from Biffi M. *et al.*, Expert Rev Med Devices 2021; 18:161–177

Study, year	Number of patients	Comparison	Mortality	HF events	AF	Main findings
**MOST, *1998*** ^28^	*n* = 2 010 (SND population)	DDD vs. VVI	=	↓	↓	Cum %VP associated to RVPIC
**DAVID, *2002*** ^29^	*n* = 506 (ICD recipients)	DDDR-70 vs. VVI-40	↑	↑		‘Unnecessary’ atrial and RV pacing are detrimental
**SAVE-PACE, *2007*** ^30^	*n* = 1 065 (SND population)	DDD + RVpm vs. DDD	=	=	↓	RVpm algorithm ↓ AF onset
**DANPACE, *2011*** ^31^	*n* = 1 415 (SND population)	DDD/R vs. AAI/R	=	=	↓	AF is related to prolonged AV interval rather than to Cum %VP
**ANSWER, 2015** ^32^	*n* = 632 (mixed population of PM recipients)	DDDR + RVpm vs. DDDR pacing	↓	↓	=	Secondary endpoints; primary endpoint similar
**MINERVA, *2019*** ^24^	*n* = 1 166 (SND population)	DDDR vs. DDDR + RVpm Baseline PR ≤ 180 ms vs. ≥180 ms			↑↓	AF is related to prolonged AV interval rather than to Cum %VP.
**CARE HF, *2009*** ^33^	*n* = 813 (CRT recipients)	CRT vs. OPT	↓	↓		Long PR is detrimental in HF patients
**REAL CRT, *2020*** ^34^	*n* = 82 (mixed population with EF ≥ 35% and PR ≥ 220 ms)	CRT vs. DDD + RVpm			↓	AF is related to prolonged AV interval rather than to Cum %VP.

AF, atrial fibrillation; AVB, atrioventricular block; CRT, cardiac resynchronization therapy; Cum %VP, cumulative percentage ventricular pacing; DDD-70, dual-chamber rate response pacing at 70 bpm; HBP, His bundle pacing; HF, heart failure; OPT, optimal pharmacologic therapy; PM, pacemaker; RVpm, right ventricular pacing minimization; RVPIC, RV pacing-induced cardiomyopathy; SND, sinus node disease; VVI-40, ventricular back-up pacing at 40 bpm.

The name of the studies are indicated as bold.

**Figure 4:**
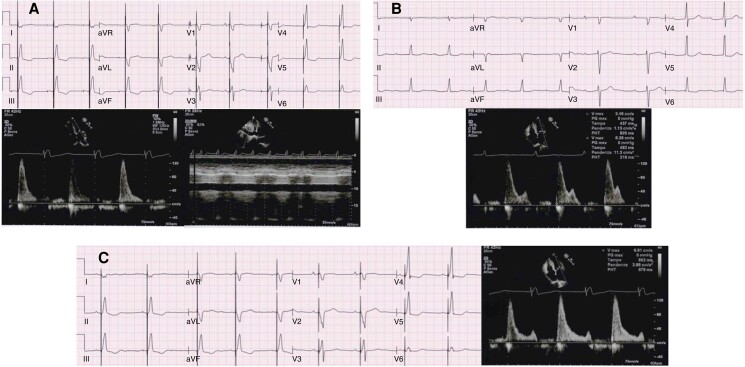
Example of a too-short PR interval in an 80-year patient with normal systolic LV function and AV block 1st/intermittent 2:1 AV block, presenting with liver congestion and swelling ankles. (A) Absence of atrial systole and restrictive filling pattern, inferior vena cava unresponsive to breathing while being DDD paced (paced AV delay 180 ms, sensed AV delay 130 ms). (B) With RVpm and lower-rate 40-bpm atrial systole occurs at a variable diastolic filling time owing to unstable PR intervals 340–400 ms. (C) At a sensed AV delay 180 m, a consistent diastolic filling time with still truncated A wave and restrictive pattern is observed, unmasking the difficulty to achieve an optimal AV coupling in aged patients.

**Figure 5:**
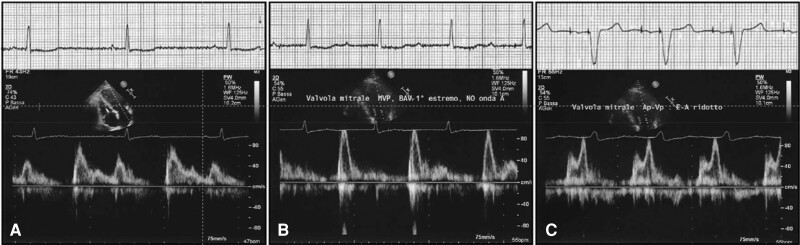
Example right ventricular pacing–induced cardiomyopathy in a SND disease patient with marked bradycardia and borderline PR interval, despite RVpm: see diastolic left ventricular filling pattern during sinus bradycardia (A). Atrial stimulation with RVpm results in an abnormally prolonged PR interval with E/A overlap and decreased LV preload (B): the patient was visited for swelling ankles and shortness of breath 6 months after implant. Tailored programming to maintain atrioventricular coupling (*C*) unveiled slightly abnormal diastolic LV function (E/A ∼ 0.7): 8 months later, the patient was hospitalized with HF and worsened LV ejection fraction at 36% due to RV stimulation. Adapted from Biffi M. *et al*., Expert Rev Med Devices 2021; 18:161–177.

**Figure 6:**
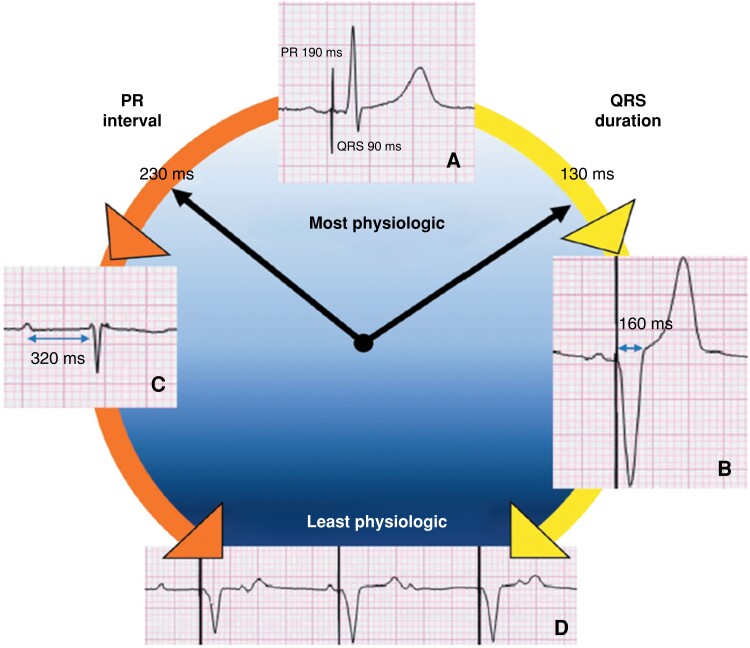
Representation of the most physiologic pacing settings as learnt from the history of cardiac stimulation. Normal atrial activation and physiologic conduction to the ventricles, as enabled by selective His bundle pacing, are preferred to ensure the best cardiac performance (A). Atrioventricular coupling with a relatively short QRS duration (130–160) by either right ventricular or biventricular pacing is a less physiologic alternative in complete heart block (B), while minimization of ventricular stimulation is a viable alternative until the 230–260 PR range when intrinsic conduction is persistent for the majority of time (C). Progressively increasing paced QRS duration (right arrow) or lengthening of the intrinsic PR interval (left arrow) promotes non-physiologic pacing and worsens cardiac function mimicking VVI stimulation, that is the least physiologic setting (D). Adapted from Biffi M. *et al*., Expert Rev Med Devices 2021; 18:161–177.

In the Minerva trial,^24^ the effect of RVpm on AF incidence was observed only in patients with a PR ≤ 180 ms, confirming that AV coupling as enabled by DDDR pacing is clinically warranted. The concept of ‘physiological’ AV interval remains difficult and should be evaluated at an individual level to avoid both the risk of a too-short (*Figure 4*) or a too-long PR interval (*Figure 5*). While there is consensus that a PR > 300 ms may cause symptoms because of a suboptimal preload,^22,27,36,37^ a PR > 230 ms marked the boundary of a non-physiologic PR interval in HF patients, who benefit from cardiac resynchronization therapy (CRT) irrespective of QRS duration and morphology.^22^ Moreover, CRT reduced new AF onset compared to RVpm in pacemaker recipients with a PR ≥ 220 ms.^34^ Thus, the knowledge of AV coupling as a ‘vulnerable’ physiological cornerstone has to be incorporated in the strategy of RVpm^39^ and dictates for careful pacemaker programming: RVpm is strongly recommended in patients with normal AV conduction but should be tailored to avoid very long PR intervals that promote an unfavourable ventricular filling (*Figure 6*). The broad adoption of conduction system pacing (CSP) will make the RVpm strategy easier in patients requiring >20% RVp owing to the possibility to achieve a physiological PR interval at no trade-off for RVp-induced cardiomyopathy (*Figure 5*).^9,40^

While several ongoing trials are comparing CSP with CRT in HF patients, the randomized Physio Vp-AF study of CSP vs. RVpm will evaluate the occurrence of persistent AF in patients with SND and a baseline PR ≥ 180 ms or with intermittent AV block 1st and 2nd, adding further knowledge to the therapeutic challenge of RVpm vs. AV coupling optimization.^41^

### Cardiac implantable electronic device–related complications and malfunctions: evaluation, troubleshooting, and practical management

Cardiac implantable electronic device (CIED)–related complications are not uncommon occurring in ≅10% of patients within 6 months of the implant.^42–45^ Most of these complications are related to the presence of transvenous leads and subcutaneous pockets. Cardiac perforation occurs in 0.4–1.2% of patients undergoing CIED implants.^42,46,47^ In a multi-centre series from the United Kingdom examining 10 631 CIED procedures, the rate of perforation was 0.5%.^48^ Overall, 98.6% of perforation presented beyond 24 h from the time of implant. The most common presenting symptom was chest pain occurring in 46% of patients. Lead electrical abnormalities were present in 86%. Tamponade was present in 17% of patients with oral anticoagulant being a risk factor. Pericardiocentesis was required in 98.6% of patients while one patient required surgical repair. In this series, all cases required lead revision. A conservative approach in certain cases (i.e. cases with normal lead parameters and a small effusion or an effusion that is drained without recurrence) might be prudent. However, these patients should be followed closely due to a higher risk of developing a significant effusion that requires an intervention. In a multi-centre series including 48 perforations (22 managed conservatively and 26 with lead revision), conservative management was associated with a higher rate of complications specifically recurrent/worsening pericardial effusion requiring drainage.^49^

Lead-related complications are also not uncommonly encountered with CIED implants.^50^ In the Danish registry, lead-related re-intervention was needed in 2.4% of patients within 6 months of implantation.^42^ In the FOLLOWPACE study, lead-related complications (excluding perforation) occurred in 5.5% of patients within 2 months of implantation.^51^ These lead-related complications remain common, so much so that the TV pacing and ICD leads are often referred to as the ‘Achilles heel’ of CIEDs.^52^

CIED infection occurs in less than 1% of new implants and is higher with generator exchange (1.5–4%) and device upgrades (2%).^42,53^ The risk of infection seems to increase with larger generators (Cardiac Resyncchronisation Therapy-Pacemake [CRT-D] and ICD > pacemaker PR interval [PPM] and Cardiac Resynchronisation Therapy-Defibrillator [CRT-P]), more complex procedures, and non-denovo CIEDs.^54^ CIED infection is associated with significant mortality, morbidity, and healthcare expenditure.^55^ Hence, prevention is paramount.

An antibacterial envelop was shown to reduce the risk of CIED infection in high-risk subgroups.^56,57^ This therapy should be considered in those patients considered at high risk of infection. Prevention of pocket haematoma is also important to reduce CIED infection. The presence of haematoma increases infection risk. In an analysis from the SIMPLE trial, the rate of peri-operative haematoma was 2.2%.^58^ The risk of infection in those patients was 10.6%. In this analysis, bridging with heparin and LMWH was associated with a 2.65-fold higher rate of haematoma formation.

### Leadless cardiac pacing

Leadless pacemakers are emerging as an alternative to traditional TV-PPM. The Micra transcatheter pacing system (TPS) (Medtronic) has been studied extensively in clinical trials.^59–61^ The Micra investigational development exemption (IDE) study enrolled 726 patients.^59^ Implant success rate was >99%, and notably, no macro-dislodgment or infections were reported in this study. However, the rate of pericardial effusion was 1.5%. The Micra post-approval registry (PAR), an FDA mandated study, enrolled more than 1 800 patients to monitor the performance of this technology in a real-world setting.^60^ The results of this study mimicked the Micra IDE results. The implant success rate was 99.1%. There was a low rate of dislodgment 0.05% and no infection requiring device removal. The rate of pericardial effusion was lower in this study (0.44% meeting major complication definition and 0.77% total pericardial effusion) as compared to the IDE. The Micra TPS clinical trials included a pre-specified comparison cohort of patients implanted with TV-PPM. Up to 63% reduction in major complications with leadless pacemaker (LP) as compared to TV-PPM was noted in these two clinical trials.

The Centers for Medicare and Medicaid Services issued a national coverage determination for LP.^61^ All Medicare patients receiving LP are automatically enrolled in a Continuous Evidence Development (CED) study. The Micra CED study compared outcomes of Medicare beneficiaries receiving a Micra device vs. those receiving a single-chamber TV-PPM. This study enrolled 5 764 patients implanted with Micra LP and 9 662 patient TV-PPM. There was no difference in 30-day complications between the two groups. The LP cohort had a higher rate of pericardial effusion as compared to the TV-PPM cohort (0.8% vs. 0.4%, *P* < 0.001) but a lower rate of device-related complications (1.4 vs. 2.6%, *P* < 0.001). When this cohort was followed for 2 years, LP were associated with 31% reduction in major complication mainly driven by 38% reduction in need for reintervention.^47^ This reduction in complications and need for reintervention was also seen in high-risk subgroups (patients on dialysis, diabetics, etc.).^62^

The AVEIR LP (ABBOTT) is the modified version of the prior ABBOTT LP (Nanostim). Unlike Micra TPS which is a tine-based fixation device, AVEIR has a helix-based fixation mechanism. The LEADLESS II Phase 2 trial showed that the implant success rate was 98%.^63^ Major complications occurred in 4% during follow-up including pericardial effusion in (1.5%), dislodgment (1%), and groin complications (1%). Recently, the result of a clinical trial testing the efficacy and safety of a dual-chamber AVEIR LP was published.^64^ The implant success rate was 98.3%. The rate of intra-procedural dislodgment was 1.7% and during follow-up (1.7%). The rate of pericardial effusion was 0.7%.

While the rate of perforation seems to be improving, some concerns remain regarding the severity of perforation with LPs. A score to predict patients’ risk of cardiac perforation has been developed and validated using the Micra TPS clinical trials data.^65^ Patients could be divided into low risk (0.4% perforation rate), intermediate risk (≅2% risk), and high risk (≅4.5%). This score could potentially be used to counsel patients regarding their risk and device choice.

The WiSE-CRT system is currently the only leadless system able to provide left ventricular pacing. It is currently still in clinical trials in the U.S. and has not received FDA approval yet. It currently has a role in failed CS upgrades and possibly non-responders to traditional CRT.^66^ The original data show a significant implant complication rate due to a high rate of arterial access complications. The transeptal approach is currently used for implantation and might be a safer option in experienced hands. Experience with a totally leadless CRT using the WiSE-CRT system has been published with encouraging results.^67^

The EHRA/HRS/LAHRS/APHRS issued a practical consideration document regarding LP.^68^ Endorsing the ESC guidelines, its use is recommended in patients with upper extremity access limitation and possibly as an alternative to traditional TV-PPM.^6^

### Lead extraction

#### What’s new concerning infections of CIED?

Device-related infection is a severe complication to CIED therapy. In the Danish pacemaker and ICD register that included 97 750 consecutive patients, the device-related infection incidence during device lifetime was 1.19% (1.12–1.26) for pacemaker, 1.91% (1.71–2.13) for ICD, 2.18% (1.78–2.64) for CRT-*P*, and 3.35% (2.92–3.83) for CRT-D.^69^

**Figure 7:**
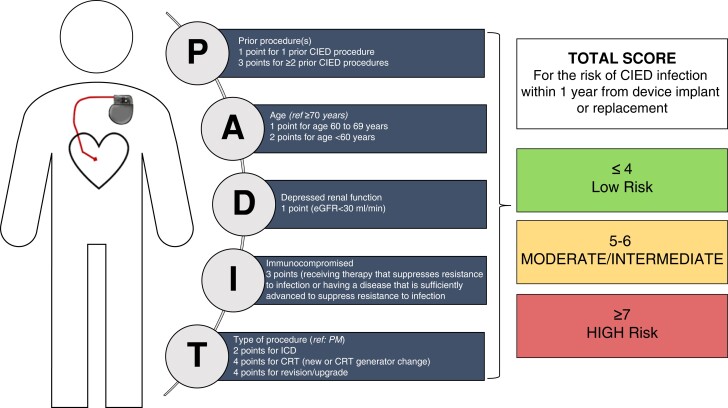
The PADIT risk score. From ref.^55^ PADIT, Prevention of Arrythmia Device Prevention Trial.

Detection of the subgroup of patients at increased risk of CIED infection is crucial, in order to take preventive measures. The PADIT infection risk score is composed of age, procedure type, renal insufficiency, immunocompromised status, and number of previous procedures (*Figure 7*).^55^ In a US data set of 54 042 index procedures among 51 623 patients with 574 infections, a one-unit increase in the PADIT score was associated with a relative 28% increase in infection risk. This score could be used in clinical practice to identify patients who may benefit from targeted interventions to reduce infection risk during implant, upgrade, or revision.^70^

As mentioned above, an antibiotic-eluting absorbable envelope (TyRX^TM^, Medtronic, Minneapolis, USA) has been developed to reduce the infection rate.^55^ One hundred and forty-four patients undergoing CIED implantation who received the antibacterial envelope were compared with a matched cohort of 382 CIED patients from a Swedish centre. The envelope group had a higher PADIT score, 5.9 ± 3.1 vs. 3.9 ± 3.0 (*P* < 0.0001). For the primary endpoint, no local infections occurred in the envelope group, compared with 2.6% in the control group (*P* = 0.04), with a more pronounced difference in the patients with a high (>7 points) PADIT score, 0 vs. 9.9% (*P* = 0.01). This study confirms the clinical efficacy and the interest of using an antibacterial envelope in the prevention of local CIED infection in patients with a higher risk guided by the PADIT score.^57^

**Figure 8:**
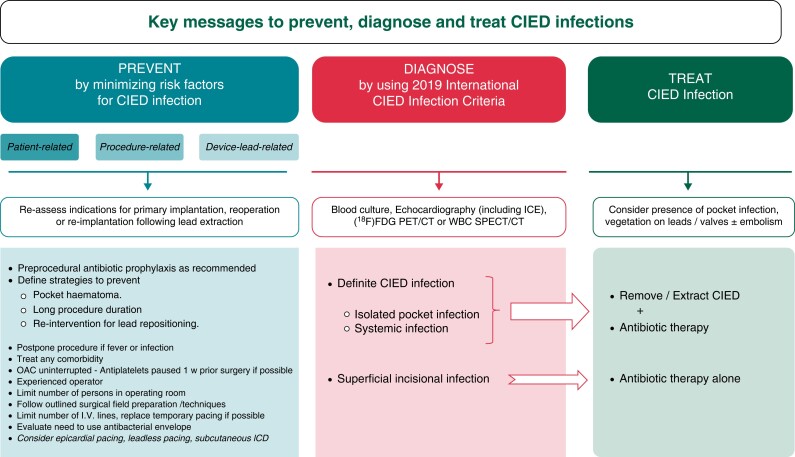
Summary of key messages for prevention, diagnosis, and management of CIED infections. From ref.^71^ CIED, cardiac implantable electronic device; [18F] FDG PET/CT, fluorodeoxyglucose positron emission tomography–computed tomography; ICD, implantable cardiac defibrillator; ICE, intracardiac echocardiography; OAC, oral anticoagulation; w, week; WBC SPECT/CT, white blood cell single-photon emission computed tomography–computed tomography.

An international consensus document on how to prevent, diagnose, and treat CIED infections has been recently released.^71^ This document gives guidance on the use of these antibacterial envelopes, but also on novel device alternatives, novel oral anticoagulants, prolonged antibiotics post-implantation, and definitions on minimum-quality requirements for centres, operators, and volumes. Many important insights are developed and delivered about all these crucial topics (*Figure 8*).^71^

#### Lead extraction indications and tools

Indications for lead extractions are well summarized in the 2018 EHRA expert consensus document.^72^ They are divided into two groups:

- Infection indications (pocket infection/erosion, lead/valvular endocarditis, bacteriaemia…)
- Non-infection indications (lead failure, abandoned lead, venous access issues, access to magnetic resonance imaging (MRI), chronic pain, recall…)

Lead removal includes a wide spectrum of tools and techniques, ranging from simple manual traction to multiple procedures and combined approaches that are also explained in this same document: superior approach, inferior/femoral approach, simple traction, locking stylets, mechanical non-powered telescoping sheaths, powered sheaths, snares, baskets, compression coils, occlusion balloons, etc.^72^

The ELECTRa study^73^ is, still currently, the largest prospective registry on transvenous lead extractions (TLE), which included a total of 3 555 consecutive patients of whom 3 510 underwent TLE at 73 centres in 19 European countries and confirmed the safety and efficacy of the current practice of TLE. Complete clinical and radiological success rates were 96.7% (95% CI 96.1–97.3%) and 95.7% (95% CI 95.2–96.2%), respectively. The primary endpoint of the in-hospital procedure-related major complication rate was 1.7% (95% CI 1.3–2.1%) (58/3510 patients) including a mortality of 0.5% (95% CI 0.3–0.8%) (17/3510 patients).^73^ TLE was associated in this registry with a higher success rate with lower all-cause complication and mortality rates in high volume compared with low-volume centres. The later paved the way for qualifications and training of operators, procedural volume, environment, and anaesthesia considerations.^72^

Longer dwelling time often requires the use of powered/mechanical sheaths for TLE. The PROMET study^74^ collected data on a total of 2 205 patients (age 66.0 ± 15.7 years) with 3 849 leads targeted for extraction in six European lead extraction centres. The median lead dwell time was 74 months. Clinical success was obtained in 97.0% of procedures, and complete extraction was achieved for 96.5% of leads. Major complications occurred in 22/2 205 procedures (1%), with a peri-operative or procedure-related mortality rate of 4/2 205 (0.18%), and minor complications in 3.1% of procedures. This study suggests that rotational TLE tools and techniques obtain similar results and can be proposed as an alternative to the laser methods.^74^

Very recently, single-centre data from 166 consecutive patients that underwent TLE requiring advanced techniques (245 leads in total, dwelling time 9.4 ± 6.3 years) have been analysed and reported.^75^ In this cohort, laser sheaths were used in 64.9%, powered mechanical sheaths in 35.1% of the procedures as primary extraction tools. The efficacy and safety of laser and mechanical sheaths were similar; however, in the subgroup of crossover procedures, mechanical tools had better performance regarding clinical success.^75^

TLE is sometimes a difficult and risky procedure requiring tool diversity and staff experience that are key for improving outcomes in the most complicated cases.

### Cardiac pacing in special populations

#### Pacing after transcatheter aortic valve implantation

Since the beginning of transcatheter aortic valve implantation (TAVI) nearly 20 years ago, injury to the conduction system necessitating pacemaker implantation showed up as a significant problem that initially involved nearly 25% of patients and is currently closer to 10%.^6, 76^ The recent ESC guidelines dedicated a full chapter to the controversy of when pacing following TAVI is indicated.^6^ The risk of fainting in an old fragile patient dictates an aggressive approach, while short-term as well as potential long-term complications with pacemaker implantation in this population are not negligible.^77,78^

**Figure 9:**
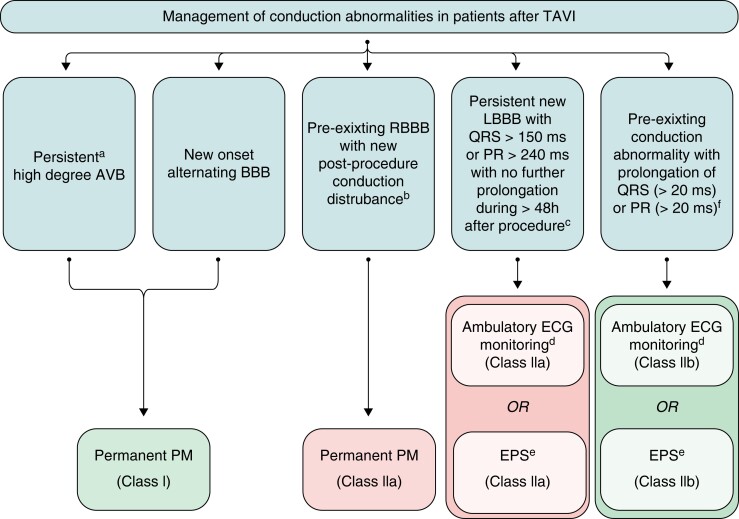
Management of conduction abnormalities after TAVI. From ref.^6^ AF, atrial fibrillation; AV, atrioventricular; AVB, atrioventricular block; BBB, bundle branch block; ECG, electrocardiogram; EPS, electrophysiology study; HV, His-ventricular interval; LBBB, left bundle branch block; LVEF, left ventricular ejection fraction; PM, pacemaker; RBBB, right bundle branch block; TAVI, transcatheter aortic valve implantation. ^a^24–48-h post-procedure. ^b^Transient high-degree AVB, PR prolongation, or axis change. ^c^High-risk parameters for high-degree AV block in patients with new-onset LBBB include AF, prolonged PR interval and LVEF < 40%. ^d^Ambulatory continuous ECG monitoring for 7–30 days. ^e^EPS with HV ≥ 70 ms may be considered positive for permanent pacing. ^f^With no further prolongation of QRS or PR during 48-h observation.

There are multiple publications on preprocedural and post-procedural risk factors and predictors of permanent pacemaker implantation following TAVI.^26,79–85^ While a lack of any conduction disturbance following TAVI carries a very low risk of development of advanced AV block and development of complete AV block that does not resolve over 24–48 h necessitates permanent pacemaker, there are many intermediate situations of an injury to the conduction system that need specific approaches including prolonged monitoring and electrophysiological conduction studies. A guideline-recommended^6^ approach is illustrated in *Figure 9*.

#### Pacing following cardiac surgery

Atrioventricular block occurs in 1–8% of cardiac operations (more common following valve operations than after coronary artery bypass) while SND may also occur in fewer operations as well as following heart transplantation.^6,86,87^ Due to the potential reversibility of post-operative block, the ideal timing for permanent pacemaker implantation has been a matter of debate over many years.^88^ The current ESC guidelines^6^ recommend a waiting period of at least 5 days with potential shortening if there is CAVB with low or no escape, with a low chance of recovery or in cases of valvular surgery with early AVB that never recovers over a 48-h observation period. In cases of endocarditis, when AVB occurs during surgery, high-risk parameter for persistent AVB exist (*Staphylococcus aureus*, intra-cardiac abscess, tricuspid involvement or previous valvular surgery). In these cases, permanent pacing should be installed immediately during surgery using epicardial approach.^89^

Tricuspid surgery forms a special group as traditional pacing involves crossing of the tricuspid valve. A mechanical tricuspid valve cannot be crossed by a pacing lead. Epicardial pacing is preferred over a lead crossing a repaired or bioprosthetic valve. When preexisting leads exist, removal and epicardial implantation are preferred over sawing the lead near the valve although the latter is not entirely contraindicated. Ventricular pacing with a preexisting bioprosthetic tricuspid valve is preferably done via coronary sinus or epicardially.^90^

For further information on pacing following heart transplantation, the reader is referred to chapter 8.2.3 in the ESC pacing guidelines.^6^

#### Pacing in congenital heart conditions

While a detailed discussion of this complex topic is beyond the scope of this publication, several principles were emphasized in the recent European guidelines for pacing.^6^ Overall, all indications in this group are based on expert opinion as there are no randomized controlled trials. An important principle is not to implant endovascular leads in the presence of intracardiac shunts. Other conditions with limited venous access may necessitate epicardial pacing. The most common aetiology of AV block in congenital heart diseases is post-operative block. Whereas in children, post-operative block usually resolves within 7–10 days (which sets the optimal time to wait before permanent pacemaker implantation), such information is scarce in adults. While patients with post-operative high-degree AV block should be paced (LOR = 1), those who had complete AV block in the perioperative period which recovered later but remained with bifascicular block may be considered for pacing (LOR = IIB). In situations of high risk for pacing in the presence of complex congenital heart disease, permanent epicardial leads should be implanted during cardiac operation.

The second important congenital situation is congenital AV block. Patients with congenital AV block should be paced if any risk factor of the following exists: symptoms, pauses > 3× the cycle length of the escape rhythm, broad QRS escape long QT complex ventricular ectopy, and daytime mean rate < 50. Some experts believe that any congenital AV block should be paced to reduce the likelihood of potentially lethal arrhythmias (IIB recommendation).

#### Pacing in hypertrophic cardiomyopathy

While RV apical pacing has been shown in several trials to modestly reduce outflow tract gradients, pacing for this indication is rarely justified.^91^ It may be considered for this purpose in patients who have another indication for pacing, in symptomatic patients who are drug refractory and cannot undergo any intervention (surgery or septal reduction) or in those undergoing septal myectomy or septal ablation with resultant AV block.

#### Rare diseases

For a detailed discussion of pacing in rare situations such as neuromuscular diseases,^92,93^ genetic cardiomyopathies (mainly Lamin AC),^94–96^ infiltrative, metabolic, and inflammatory disease, including the decision among pacemaker and ICD implantation, please refer to the guideline document.^6^

### CIED management in complex clinical scenario

#### MRI environment

The management of implantable devices in an MRI environment has long been discussed in the literature^97–100^ and is best covered recently by an EHRA document on magnetic interference.^101^ The potential effects of MRI on CIEDs include triggering of asynchronous pacing thus resulting in atrial or ventricular arrhythmias, heating of the heart tissue surrounding leads resulting in change in electrical parameters, and reprogramming of the device including power on reset and signs of battery depletion. Oversensing is most common and may result in pacing inhibition and/or inappropriate ICD therapies if not pre-programmed. Overall, the incidence of significant complications with appropriate programming is very low and clear recommendations exist about preparation and pre-programming of patients with devices before MRI. All these recommendations refer to patients several weeks or more following implantation; recent implantation is considered a relative contraindication.

Abandoned leads, epicardial leads, and adapters are considered contraindications to MRI due to the lack of information and theoretical arguments but have been shown by small series not to cause any troubles.^102^ The current wide availability of MRI conditional devices makes the procedure much simpler and safe. Nevertheless, the availability of device-competent staff and emergency routines in the MRI suite are still necessary.

**Figure 10:**
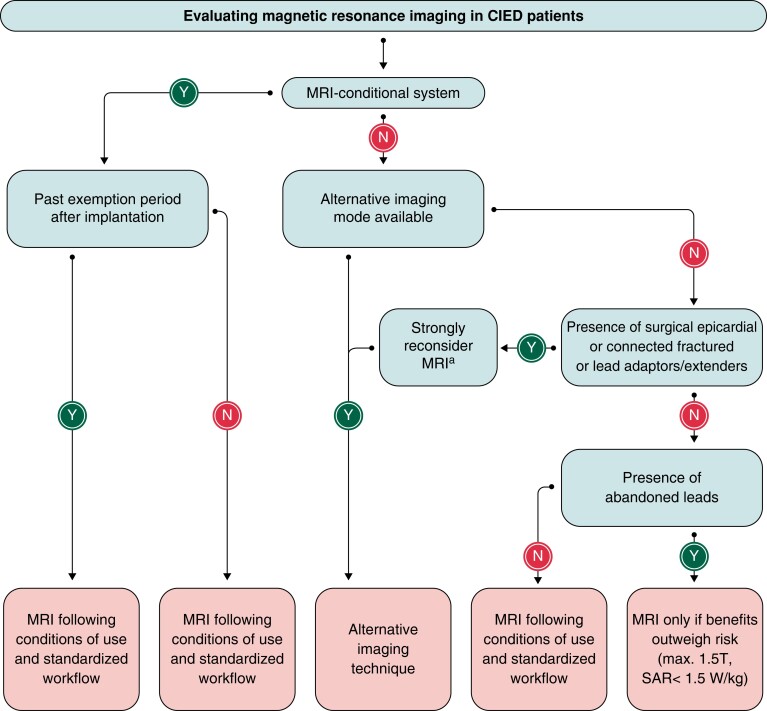
Flowchart for evaluating MRI in CIED patients. From ref.^101^ MRI, magnetic resonance imaging; SAR, specific absorption rate. ^a^Consider only if there is no imaging alternative, and the results of the test is crucial for applying life-saving therapies for the patients.

**Figure 11:**
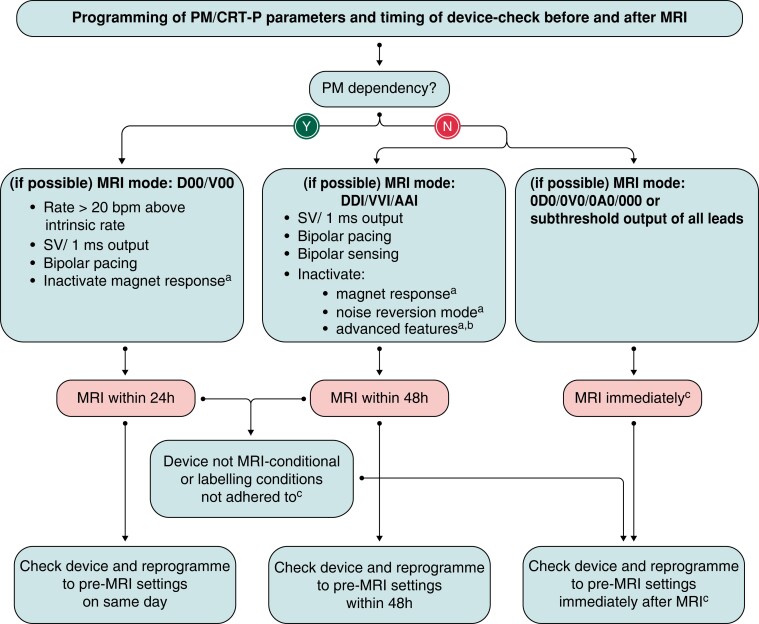
Programming of device parameters and timing of device check before and after MRI. From ref.^101^ AF, atrial fibrillation; MRI, magnetic resonance imaging. ^a^If available. ^b^Rate hysteresis; atrial anti-tachycardia pacing; premature ventricular complex and premature atrial contraction triggered pacing; AF therapies–rate smoothing; overdrive pacing; conducted AF response. ^c^In CIED with automatic MRI mode activation, the scan may be performed electively after the pre-scan follow-up and reprogramming after the intervention may not be necessary.

*Figures 10* and *11* summarize the EHRA approach to MRI with devices.^101^ Further details on specific programming and follow-up are available in the EHRA document.^101^

#### Perioperative management of implantable devices

**Figure 12:**
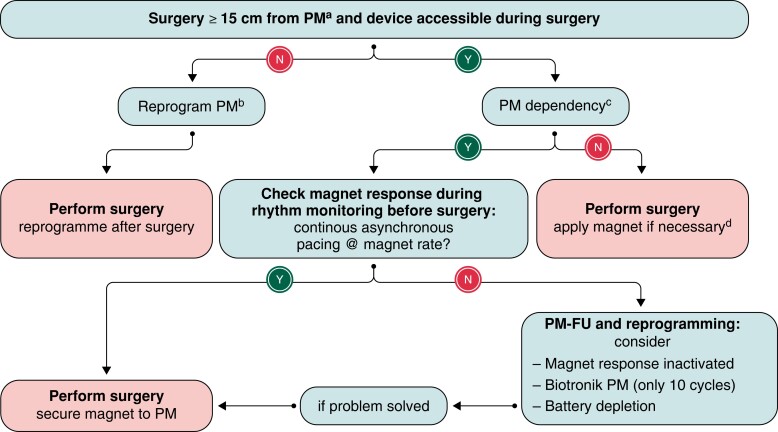
Algorithm for perioperative management of PM (including CRT-P) during surgery. From ref.^101^^a^Reprogramming/magnet application is optional, if surgery is performed below the iliac crest and no full-body return electrodes are used; ^b^asynchronous mode (D00/V00/A00); rate response may be inactivated to avoid rapid pacing with patient mobilization or respiratory monitoring (if the PM has a minute-ventilation sensor); ^c^absence of intrinsic escape rhythm or heart rate, 50-bpm causing symptoms; ^d^asystole or haemodynamically relevant bradycardia during electrocautery.

This topic is also thoroughly reviewed in EHRA consensus paper on electromagnetic interference (EMI) with practical recommendations, some of which are innovative.^101^ The principal risk of surgery is EMI caused by cautery (mainly unipolar), and it is relevant mostly in procedures performed above the umbilicus. Notably, magnet use during surgery, which used to be discouraged in the past due to illusive reprogramming of devices with open reed switch, is now recommended if needed during surgery. Safe taping of the magnet over the device is recommended. Magnets are used to prevent oversensing inhibition of pacemakers in dependent patients or detection of the cautery by defibrillators resulting in inappropriate therapy delivery. Magnet can be used if the operative field is not too close (15 cm) to the device. When the field is close, then, reprogramming of the device is necessary before surgery if the patient is pacemaker dependent or has an ICD. *Figure 12* illustrates the EHRA-recommended approach to management of devices during surgery.^101^

#### Radiotherapy in the presence of implantable devices

Over the years, 2–7% of patients with CIEDS undergoing therapeutic radiation developed some kind of device malfunction.^101,103,104^ The risk of malfunction is related to the location and cumulative dose of irradiation, mainly at the generator site, type of energy (proton beam more dangerous), and modes of shielding.^105^ Until recently, there was wide variation in the approach to patients with CIEDS undergoing radiation therapy^106^ and the EHRA document meant to set more uniform standards.^101^

The main effect of radiation on CIEDs is in damaging device memories, causing temporary or permanent programming change, and rarely EMI during the irradiation session. The damage is cumulative and may develop late rather than early in the course of radiation sessions. Operations to remove the pacemaker to a different place are very rarely necessary these days and mainly done if the CIED interferes with effective energy delivery to the tumour site.

**Figure 13:**
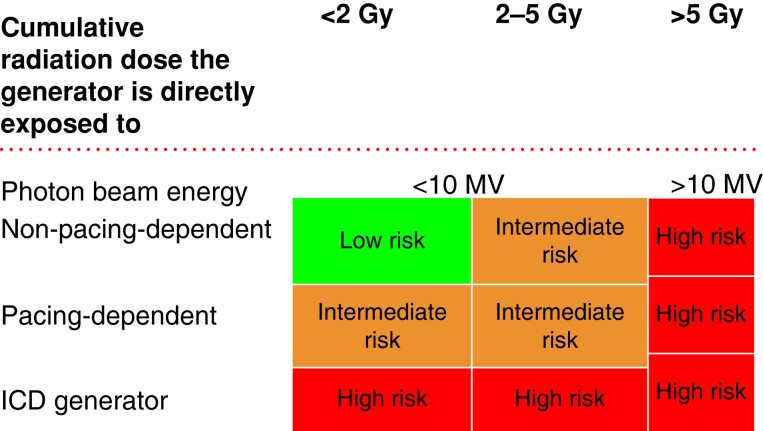
Risk stratification for CIED malfunction. From ref.^101^

Risk stratification is needed prior to radiation therapy. This is based on the radiation dose for the device, type of energy (photon beam?), pacemaker dependency, and the presence of an ICD (*Figure 13*). All patients with CIEDS have to be monitored at least vocally during the session, and a code cart should be available. CIED-trained professionals should be available in the hospital.

In the low-risk group, the device has to be interrogated prior to and after completion of all radiotherapy fractions. In the intermediate-risk group devices, interrogation should take place as above but also in the middle of the period. ECG monitoring is mandatory in any case of suspicion of device malfunction during sessions of radiation. Remote monitoring is also valuable.

In the high-risk group, remote monitoring or weekly interrogation is recommended. All other aspects are unchanged and ECG monitoring is also mandatory during session. Most cases do not have to be reprogrammed for the irradiation. For more detailed discussion of this topic, please refer to EHRA document.^101^

### Future perspectives in cardiac pacing

The evolution of cardiac pacing has progressed through several eras that include the development of the first implantable permanent pacemakers, dual-chamber pacing, advanced programming, cardiac resynchronization therapy, remote monitoring, leadless pacing, and finally physiologic pacing. Physiologic pacing has evolved rapidly through a variety of anatomic targets including the His bundle, the left bundle branches, and now the right bundle branch.^107^ Innovation in cardiac pacing is more intense and diverse than ever before. The development of physiologic pacing has been one of the most notable advances in pacing in the several decades. While tremendous progress has been made, the field of physiologic pacing remains in its infancy. Left bundle branch area pacing is now the dominant and most reproducible form of physiologic pacing,^108^ but how that is optimally combined with other pacing technologies is largely unknown. We still don’t have pacemaker generators designed to deliver physiologic pacing nor do we know what combination of physiologic and resynchronization technologies result in optimal treatment (and prevention) of HF.^109^ While the longevity of pacemaker batteries has improved over the years, pacemaker battery innovation has been characterized by relatively small, incremental steps in battery chemistry. Rechargeable pacemakers would avoid many of the challenges associated with the need for repeated generator replacements. While the external application of electromagnetic induction currents to recharge pacemakers was reported in 1965,^110^ rechargeable technologies have not entered clinical practice. However, the future of pacing will likely include not one but several rechargeable battery technologies. Advances in both external charge technologies and self-recharging devices have the potential to accelerate the development and the utility of these systems. Self-recharging devices that harvest *in vivo* biomechanical energy including through the use of triboelectric nanogenerators have exciting possibilities.^111^ The development of leadless pacing has also been a notable advance in pacing. Within a decade, leadless pacing has evolved from single-chamber VVI/R devices, to single-chamber VDD pacing, and now dual-chamber pacing. Modular dual-chamber leadless pacing has entered clinical trials, and the early results are very promising with 97% of patients achieving ≥70% atrioventricular synchrony.^64^ Totally leadless cardiac resynchronization therapy has been demonstrated to be effective with leadless RV pacemakers paired with leadless ultrasound-based endocardial left ventricular pacing.^67^ Integration of leadless technologies across indications and across device platforms will continue to evolve. As more and heterogeneous pacing technologies enter clinical practice, selection of pacing systems for specific patients will also become more complex. Personalization of pacing therapy will be more important than ever. The COVID pandemic highlighted the value of remote and virtual care. Future advances in pacing will also include further adaptions that facilitate more patient-centred care that is more convenient and accessible. Technologies for remote programming are being developed and have the potential to change care dramatically, potentially removing the need for most in-person visits.^112^ Personalized approaches to pacing will evolve in the future, especially as machine learning and artificial intelligence are applied predictive analytics. For example, AI-assisted analysis of ECGs may help identify patients who would benefit from permanent pacing before they develop symptoms from conduction disorders. Such techniques have already been used to predict who requires pacemaker implantation after TAVI.^113^ Improved pacemaker diagnostics and their analysis will also allow for improved personalized care. One notable example is the ability of device diagnostics to identify patients who may have sleep apnoea.^114^ Personalized medicine has been a challenge for medical therapy, but device therapy may be able to deliver on this promise more effectively, particularly due to synergies in innovation in how we identify who needs pacing and when (i.e. AI-based prediction using ECGs), how we provide pacing (i.e. remote analysis and programming), and how we use the information we gather from pacing (i.e. identification of sleep apnoea).
